# Ageing Together: Interdependence in the Memory Compensation Strategies of Long-Married Older Couples

**DOI:** 10.3389/fpsyg.2022.854051

**Published:** 2022-04-01

**Authors:** Celia B. Harris, John Sutton, Paul G. Keil, Nina McIlwain, Sophia A. Harris, Amanda J. Barnier, Greg Savage, Roger A. Dixon

**Affiliations:** ^1^MARCS Institute for Brain, Behaviour, and Development, Western Sydney University, Penrith, NSW, Australia; ^2^Department of Psychological Sciences, Macquarie University, Sydney, NSW, Australia; ^3^Institute of Ethnology, Czech Academy of Sciences, Prague, Czechia; ^4^Discipline of Psychiatry and Mental Health, University of New South Wales, Sydney, NSW, Australia; ^5^Department of Psychology, University of Alberta, Edmonton, AB, Canada

**Keywords:** memory compensation, couples, cognitive ageing, memory compensation questionnaire, interdependence, transactive memory

## Abstract

People live and age together in social groups. Across a range of outcomes, research has identified interdependence in the cognitive and health trajectories of ageing couples. Various types of memory decline with age and people report using a range of internal and external, social, and material strategies to compensate for these declines. While memory compensation strategies have been widely studied, research so far has focused only on single individuals. We examined interdependence in the memory compensation strategies reported by spouses within 58 older couples. Couples completed the Memory Compensation Questionnaire, as well as an open-ended interview about their memory compensation practices. We found that internal, intra-individual memory compensation strategies were not associated within couples, but external, extra-individual strategies showed interdependence. Individuals’ scores on material/technological compensation strategies were positively correlated with their partners’. Reported reliance on a spouse was higher for men and increased with age. Our open-ended interviews yielded rich insights into the complex and diverse resources that couples use to support memory in day-to-day life. Particularly evident was the extent of interaction and coordination between social and material compensation, such that couples jointly used external compensation resources. Our results suggest that individuals’ reports of their compensation strategies do not tell the whole story. Rather, we propose that older couples show interdependence in their memory compensation strategies, and adopt complex systems of integrated material and social memory compensation in their day-to-day lives.

## Introduction

The human experience involves developing and ageing, living and working, within various social groups. Older couples who have spent decades sharing their day-to-day lives are a paradigm case of a close social group who develop and age together. There is increasing recognition that the cognitive changes that come with ageing impact on both members of a couple, and are productively viewed, understood, and treated in that context (Dixon, 1999; Ingersoll-Dayton et al., 2013; Harris et al., 2014; Scherrer et al., 2014; Hoppmann and Gerstorf, 2015; Wadham et al., 2016). There is emerging evidence that the cognitive trajectories of ageing couples are related (Gerstorf et al., 2009) and that close partners have a particular ability to prompt and facilitate memory performance, in both healthy ageing and Alzheimer’s dementia (Kemper et al., 1995; Rauers et al., 2011; Harris et al., 2017). The assessment and interpretation of individual cognitive competence and change as people age can therefore benefit from considering a full range of changing contexts. This particularly includes specific characteristics of ageing individuals’ broader social and material context—the people, tools and artefacts that are part of the everyday environment—which may serve as external resources that scaffold and support everyday memory performance (Dixon, 2011). Such tools and practices are likely relied on across the lifespan (e.g., Nelson and Fivush, 2004; Habermas et al., 2010; Hirst et al., 2018; Soares and Storm, 2021), but may have particular value as people can use these tools to compensate for cognitive changes associated with older age (Dixon, 2011; Harris et al., 2014). In the current manuscript, we examine possible interdependence in the everyday memory compensation strategies used by individuals within couples; do couples co-ordinate their social and material memory compensation strategies?

### Memory Compensation Strategies in Ageing

The possible benefits of both material and social memory supports in ageing have long been recognized in models of “memory compensation” in gerontology research (Bäckman and Dixon, 1992). Memory compensation encompasses a range of intra-individual and extra-individual strategies by which changes in cognitive ability can be mitigated, enabling failing memory to be improved (Bäckman and Dixon, 1992; Dixon and Bäckman, 1995; Dixon et al., 2001). Theories have examined different mechanisms for memory compensation. With remediation strategies, people can boost their failing remembering processes using strategies such as taking more time or making more effort to increase performance. With substitution strategies, people can substitute their fallible cognitive capacities with new different internal processes like mnemonic tricks and deliberate retrieval practice, or by outsourcing to more reliable external resources. External resources include both material resources (like a diary, or an iPhone) and social resources (like a spouse; Dixon et al., 2001).

The Memory Compensation Questionnaire (MCQ; Dixon et al., 2001, 2003; Dixon and de Frias, 2004, 2007; de Frias and Dixon, 2005) indexes individuals’ awareness and everyday use of a variety of approaches and strategies to “compensate” for perceived or actual memory performance decline. The MCQ was developed and refined by surveying the everyday memory practices reported by large numbers of older adults (Dixon et al., 2001). It has five subscales, covering substitution (External, Internal, and Reliance) and remediation strategies (Time and Effort), both intra- and extra-individual (see Table 1 for full details). The MCQ has shown confirmatory factor structure (validating the subscales), measurement invariance (over time), long-term stability/reliability, predictive validity, clinical differences, and several sources of variability (De Frias et al., 2003; Dixon et al., 2003; Dixon and de Frias, 2004, 2007, 2009; de Frias and Dixon, 2005).

**TABLE 1:** Classification of subscales in the Memory Compensation Questionnaire.

Subscale type	Mechanism	Subscale label	Locus	Subscale description	Items
Strategy	Substitution	External	Extra-individual	Use of external, material tools and resources	8
		Internal	Intra-individual	Use of mnemonics or internal strategies	10
		Reliance	Extra-individual	Use of another person as a memory resource	5
	Remediation	Time	Intra-individual	Investing more time in encoding	5
		Effort	Intra-individual	Investing more effort in encoding	6
General		Success	N/A	Concern with accuracy in memory performance	5
		Change	N/A	Perceptions of increased strategy use in recent years	5

Research on the memory compensation strategies reported by ageing adults has found that the use of material external aids is the most endorsed strategy, and somewhat surprisingly, that reliance on social support is the least endorsed (Dixon et al., 2001). Notably, there are a range of individual difference factors that influence older adults’ reported memory compensation strategies, including factors related to social integration and health (De Frias et al., 2003). Particularly, although it is the least endorsed strategy, reported reliance on social support increases with age—particularly for men—and with cognitive decline (Dixon et al., 2001). People with more subjective memory complaints and poorer verbal learning ability also report more social reliance strategies, suggesting that other people become an increasingly important source of memory compensation as individual abilities decline (Lin et al., 2020).

### Cognitive Interdependence in Couples

So far, memory compensation strategies have been examined within individuals. However, the memory compensation strategies adopted by an individual may depend critically on what their partner does, a factor that has not yet been studied. The key role of partners is supported by “transactive memory” theory (Wegner et al., 1985, 1991; Wegner, 1987, 1995), in which people in close relationships are argued to develop cognitive interdependence, such that they are more accurately considered as a system or a single unit of analysis rather than independent individuals [see also Harris et al. (2014) and Barnier et al. (2018a)]. According to transactive memory theory, people living and working together in groups of two or more over time develop a pattern of coordination on joint cognitive tasks, whereby their individual memory systems share the encoding, storage, and retrieval of information. Transactive memory systems are potentially more efficient and effective compared to individuals remembering in isolation. Cognitive load is distributed among group members and redundancy can be limited through the development of individual expertise. Differentiated cognitive processes are coordinated and function in parallel, as group members simultaneously perform complementary tasks to solve joint problems. When memory performance on shared tasks overlaps and interacts, it can produce outcomes that are distinct in process, quality, or quantity from what is produced when people remember alone (Harris et al., 2014; Barnier et al., 2018a).

Consistent with transactive memory theory, prior research has demonstrated that older couples can benefit from remembering together, such that they recall more when together than they remember separately (Gould and Dixon, 1993; Dixon and Gould, 1998; Johansson et al., 2005; Harris et al., 2017; Barnier et al., 2018b). The pattern of “collaborative facilitation” observed in older couples, particularly when recalling personally relevant information (Barnier et al., 2018b) contrasts with a large literature on “collaborative inhibition,” whereby pairs of strangers typically perform worse when remembering together than when recalling separately (Harris et al., 2008; Marion and Thorley, 2016). Older couples may therefore have a special ability to remember effectively together in ways that other kinds of groups do not.

We note that it is not yet known whether younger couples experience similar benefits, and what timeframe of relationship is sufficient to develop an effective transactive system. Barnier et al. (2014) did not find benefits of collaboration for younger couples in their study of older and younger couples recalling autobiographical events, and Gould and Dixon (1993) found that younger and older married couples collaborated in quite different ways when recalling a vacation, but did not compare to individual performance. Gagnon and Dixon (2008) found some benefits for both younger and older adults of collaborating with their intimate partner (compared to a stranger) and some of these effects were more marked for long-married older couples. Taken together, these suggest that effective joint remembering may develop with time and joint experience as a couple, or may be particularly evident when cognitive support is needed. However, these possibilities have not been definitively investigated, with extant studies varying in methodology, comparison, and outcome measures, and no research yet that distinguishes age from length of relationship [for discussion see Gagnon and Dixon (2008) and Dixon (2011)]. Further, the collaborative benefits identified for older couples in prior research show individual differences, such that not all older couples collaborate successfully. Instead, benefits depend on the successful use of communication techniques such as cuing and acknowledging expertise during the joint task (Gould et al., 1994; Harris et al., 2011, 2019; Browning et al., 2018). These findings suggest that older couples are able to use effective communication to coordinate their remembering and boost each others’ performance on memory tasks.

Transactive memory theory therefore predicts that people who live and age together, collaboratively grappling with the shared experiences of daily life, learn to undertake cognitive tasks in relation to the presence and actions of their partner. Who best encodes and recalls what, and how partners coordinate during the act of remembering together, are skilled practices negotiated and developed across time in long-term relationships. In some circumstances, partners may have developed similar memory organization, or “integration” (c.f. Wegner et al., 1985), a quality beneficial for meaningful autobiographical remembering about shared life experiences. Alternatively, systems defined by specialization or “differentiation” (c.f. Wegner et al., 1985) may arise due to a variety of factors, including different types of tasks, the personality dynamics in the relationship, the norms of gender roles, or the need to compensate for the decline in cognitive function of one partner (e.g., Grysman et al., 2020). Crucially for the current research, interdependence predicts that the memory compensation strategies of individuals within couples may vary together in systematic ways (Dixon, 1999, 2013), such that one individual’s cognition and behaviour is at least partly explained by the cognition and behaviour of the other. We therefore examined relationships between the memory compensation strategies adopted by men and women within long-standing couples.

### Social and Material Forms of Memory Compensation

Transactive memory theory motivates a focus on extra-individual strategies, both social and material. Wegner’s original conceptualization focused on the social resource of interpersonal relationships, specifically intimate couples, but the concept of transactive memory has since been linked to material tools such as devices that access the internet (Sparrow et al., 2011; Heersmink and Sutton, 2020). In studies of memory compensation, material resources such as notebooks and calendars are considered separately from social resources such as a spouse or friend. However, in everyday contexts, the distinction between social and material resources is not so simple, and in many settings human cognitive processes involve complex entanglements with both and material aspects of the environment [see Barnier et al. (2008); Harris et al. (2014), and Hutchins (2014)]. Ageing individuals in long-term relationships may coordinate their shared and discrete activities by—separately or together—using external resources like calendars and diaries to structure their routines and support their memories. In the current research, we examined whether and how social and material memory compensation strategies might interact with one another.

### The Current Study

In the current study, we investigated self-reported use of social and material resources in older, long-married couples, using the Memory Compensation Questionnaire as well as a semi-structured interview. We examined how memory compensation might be interdependent in older couples. We looked for three kinds of evidence of cognitive interdependence. First, we analysed participants as individuals. Does this particular population—all members of longstanding intimate partnerships—report higher use of social compensation strategies than previously reported in studies of the general older population, not all of whom might have an intimate partner to rely upon? Second, we assessed whether there were interrelationships between the compensation strategies reported by individuals within couples. Given that couples share their cognitive trajectories, as well as their day-to-day life and domestic arrangements, we hypothesized that partners’ reported compensation strategies would be correlated. We did not have *a priori* hypotheses about the nature of this relationship. A positive correlation between partners on the same subscales would indicate similarity or integration in memory compensation strategies. A negative correlation between partners on the same subscales would indicate differentiated memory compensation strategies. Other patterns of intercorrelation across different subscales would indicate other kinds of complementarity in memory compensation strategies. Third, we used a semi-structured interview to catalogue and classify couples’ external memory resources, in order to examine how material resources were integrated within the social couple-system. We aimed to characterize the complex and dynamic memory compensation strategies that older couples employ in their everyday life, encompassing both material and social memory supports.

## Materials and Methods

### Participants

Participants were 116 older adults (58 women and 58 men), ranging in age from 68 to 90 years (*M* = 75.54, *SD* = 5.21). Men (*M* = 76.91, *SD* = 5.33) were significantly older than women (*M* = 74.17, *SD* = 4.75) by an average of just under 3 years, *t*(114) = 2.92, *p* = 0.004. These 116 individuals made up 58 male-female, long-term couples, married for between 13 and 65 years (*M* = 49.91, *SD* = 9.16) and living together independently in their homes. We recruited participants in two ways, as part of two other studies reported elsewhere. Nineteen couples were recruited *via* a local community organization in Sydney, Australia, as part of their participation in another study [see Harris et al. (2017)]. Thirty-nine couples were recruited *via* the Australian Imaging, Biomarkers and Lifestyle (AIBL) Study of Ageing in Melbourne, Australia as part of their participation in another study (Barnier et al., 2018b; Harris et al., 2019). For the first sample, all participants were living independently in the community as a couple and had not received any clinical diagnosis implicating memory problems. For the second sample, they were identified by AIBL as “healthy controls” indicating normal cognitive function on neuropsychological screening undertaken as part of that large longitudinal study. Participants recruited *via* the community were paid AU$50 each for their participation in a broader study, while participants recruited *via* AIBL were not remunerated. There were some demographic differences between samples in terms of age: men recruited *via* the community were older on average than those recruited *via* AIBL, *t*(114) = 2.42, *p* = 0.017, and this was marginally the case for women as well, *t*(114) = 1.98, *p* = 0.053. The length of relationship was not significantly different, *t*(114) = 0.63, *p* = 0.528. Because of age differences between genders and samples, we included age as a covariate in analyses reported below. For the first sample, we did not obtain information about education level or conduct a measure of cognitive function. For the second sample, participants had between 6 and 23 years of formal education (*M* = 14.47, *SD* = 4.11) and they scored between 23 and 30 (*M* = 28.88, *SD* = 1.43) on a Mini Mental State Examination conducted on the day of testing.

### Materials

#### The Memory Compensation Questionnaire

The Memory Compensation Questionnaire (MCQ; Dixon et al., 2001; de Frias and Dixon, 2005) is a 44-item survey designed to measure the variety and extent of ways in which an individual compensates for memory losses and impairments. The MCQ shows stability in scores over a 3-year period (Dixon et al., 2001). Self-reports are collected across seven subscales; five are “strategy subscales” relating to efforts made to compensate for memory loss; two are “general scales” which gauge the awareness of memory changes and level of commitment to memory performance (de Frias and Dixon, 2005). The five strategy subscales cover both substitution and remediation strategies. Because of our current focus on external memory supports, we also classified each strategy as “intra-individual” or “extra-individual,” noting that the MCQ differentiates between two sources of extra-individual support: material resources and social resources. Table 1 provides the subscales and their descriptions that address each memory compensation mechanism, as well as the number of items in each subscale.

Participants rated items such as: “Do you post notes on a board or other prominent place to help you remember things for the future (for example, meetings or dates)?” (External); “Do you use letters as cues (in other words, go through the alphabet) when you want to remember the name of a person, a city, or something else?” (Internal); “Do you sometimes ask someone (for example, spouse or friend) to help you remember when you are going to start a trip?” (Reliance); “Do you take your time to go through and reconstruct an event you want to remember?” (Time); “When you want to remember a story, do you read it more than once?” (Effort); “When you want to remember a newspaper article, is it important to you to remember it perfectly?” (Success); and “Do you use such aids for memory as notebooks or putting things in certain places more or less often today compared to 5–10 years ago?” (Change). They rated 44 total items on 5-point Likert scales (1 = *never*, 5 = *always*). The overall reliabilities for the subscales as measured by Cronbach’s alpha were acceptable, ranging from 0.65 to 0.83.

### Procedure

All participants completed a “pencil and paper” version of the MCQ within the context of a number of other individual and collaborative memory tasks, including word list recall, personal information, autobiographical memory, and a range of questionnaires and neuropsychological measures. These memory measures are reported elsewhere (Harris et al., 2017, 2019; Barnier et al., 2018b).

We conducted interviews with a subset of 48 couples (9 of those recruited from the community, and all 39 of those recruited *via* AIBL), at the end of the experimental session. We asked couples to describe their everyday remembering practices, tools, and strategies, how they remember appointments and events in day-to-day life, their diaries, calendars, and other external memory resources, and any division in responsibility for the checking and maintenance of such resources. The opening question was, “Can you tell us about how you remember together?” Further specific questions included, “What kinds of things do you remember in your day-to-day life, and how do you remember these things?”; “When do you look at your resources?”; “Who looks at your resources?”; “Who updates your resources?”; “Where are your resources kept?”; “How do you help each other remember?”; “Are there things one person is better at remembering?” The same core set of questions were asked of each couple, and the interview lasted approximately 15–20 min.

### Interview Coding

Our aim in the interviews was to determine how couples used material and social resources in their everyday life, and how they coordinated remembering and memory compensation strategies between them. To meet these aims, we counted the number of material resources reported by each couple, and for each resource, scored whether or not it was digital or physical, what kind of memory task it supported, whether the ownership, entry and the use of information was shared or individual, and whether one individual benefited vicariously from the other’s resource. All 48 interviews were double coded by two independent coders, and overall interrater reliability was high at 88.6%. All disagreements were discussed and resolved by the coders to produce a final set of agreed codes.

We used NVivo 12 (QSR International) qualitative data analysis software to organise and analyse the interview data. We examined the transcripts for emergent themes relating to couples’ use of memory resources, and identified common themes relating to function, coordination, responsibility, vicarious benefit, reliance, and age-related memory decline, around which we organise our results.

## Results

### Scores on Subscales

First, we were interested in which compensation strategies were most highly reported by participants. We conducted a one-way repeated measures ANOVA on individuals’ MCQ scores with the within-subjects factor of subscale (external vs. internal vs. time vs. reliance vs. effort vs. success vs. change). Values reported here are Greenhouse-Geisser statistics, with the degrees of freedom corrected due to a violation in the assumption of sphericity. This analysis yielded a significant main effect of subscale, *F*(6,106) = 93.91, *p* < 0.001, η_*p*_^2^ = 0.46. Overall, External strategies received the highest ratings and Reliance strategies received the lowest ratings (all *p*s < 0.001), with the other subscales somewhere in between (see Table 2).

**TABLE 2:** Scores on MCQ subscales, reported by participant gender.

Subscale	Overall	Men	Women
Internal strategies	2.99 (0.47)	2.94 (0.50)	3.04 (0.43)
Time strategies	2.83 (0.53)	2.81 (0.54)	2.85 (0.53)
Effort strategies	3.47 (0.55)	3.41 (0.54)	3.52 (0.56)
External strategies	3.99 (0.69)	3.86 (0.67)	4.12 (0.69)
Reliance strategies	2.51 (0.67)	2.73 (0.67)	2.30 (0.61)
Concern with success	2.95 (0.70)	2.84 (0.67)	3.07 (0.71)
Perception of change	3.31 (0.43)	3.27 (0.38)	3.35 (0.48)

*Scores are means across all items belonging to each subscale, rated on a 5-point scale from 1 = never to 5 = always. Values in parentheses are standard deviations.*

### Individual Differences in Compensation Strategies

#### Age

Consistent with prior research, results indicated a significant but small positive correlation between age and scores on the Reliance subscale, *r* = 0.26, *p* = 0.005. None of the intra-individual subscales (Internal, Time, Effort) nor the External subscale were significantly associated with age, all *r*s < 0.081, all *p*s > 0.393. Finally, there was no significant relationship between age and the Change or Success subscales, all *r*s < 0.08, all *p*s > 0.43. Overall, older participants reported more social compensation strategies of relying on other people.

#### Gender

To examine whether there were gender differences across subscales, we conducted a 2 (gender: male vs. female) × (5) (subscale) mixed ANOVA on the individual responses, and included participant age as a covariate since the men were significantly older than the women. This analysis yielded no significant main effect of gender, *F*(1,109) = 0.34, *p* = 0.564, nor of subscale, *F* = *F*(4,436) = 2.51, *p* = 0.056, η_*p*_^2^ = 0.02, but there was a significant interaction between gender and subscale, *F*(4,436) = 5.96, *p* < 0.001, η_*p*_^2^ = 0.05. There were no main or interaction effects of participant age, all *F*s < 1.19, *p*s > 0.314.

Planned pairwise comparisons between men and women on each subscale, including age as a covariate, indicated that there were no gender differences on the intra-individual subscales (Internal, Time, Effort), all *F*s < 1.49, all *p*s > 0.23. For the extra-individual subscales, there were significant gender differences, such that women scored significantly higher than men on the External subscale, *F*(1,109) = 4.49, *p* = 0.036, η_*p*_^2^ = 0.04 and men scored significantly higher than women on the Reliance subscale, *F*(1,109) = 8.51, *p* = 0.004, η_*p*_^2^ = 0.07, even with age as a covariate. Finally, there were no significant gender differences on either the Change or Success subscales, all *F*s < 2.71, all *p*s > 0.10 (see Table 2). Age had a significant effect on Reliance, *F*(1,109) = 4.21, *p* = 0.043, η_*p*_^2^ = 0.04, but not on any other subscales, all *F*s < 0.46, all *p*s > 0.50, consistent with the correlations reported above.

To further investigate reasons for gender differences in patterns of memory compensation, we obtained correlations between scores on the Success subscale and the five Compensation subscales, separately for men and women. For men, increased concern with memory success was associated with an increased use of Internal strategies, *r*(56) = 0.34, *p* = 0.011 and Effort strategies, *r*(56) = 0.35, *p* = 0.009, as well as Reliance strategies, *r*(56) = 0.30, *p* = 0.027. There was a weaker non-significant relationship with Time strategies, *r*(56) = 0.25, *p* = 0.066, and no relationship with External strategies, *r*(56) = 0.07, *p* = 0.624. For women, increased concern with memory success was associated with increased Effort strategies, *r*(56) = 0.31, *p* = 0.020 and marginally with Internal strategies, *r*(56) = 0.26, *p* = 0.051, but not with Reliance, Time, or External strategies, all *r*s < 0.11, all *p*s > 0.43. Together, these results suggest that when the men in our sample perceived a need for memory support, they compensated by relying on their wives as well as by using intra-individual strategies, but women did not report a similar reliance on their husbands, relying instead only on intra-individual strategies.

### Within-Couple Relationships

Most importantly for the aims of the current paper, within couples we obtained correlations between husbands’ and wives’ scores on each strategy. The use of External strategies was strongly and significantly correlated between partners, *r*(56) = 0.71, *p* ≤ 0.001, and there was also a weak non-significant relationship for Internal strategies, *r*(56) = 0.23, *p* = 0.086, with no significant correlations within couples for Time, Effort, or Reliance strategies, all *r*s < 0.15, all *p*s > 0.27. That is, people who reported higher use of External strategies had partners who reported the same.

We conducted an exploratory Principal Component Analysis (PCA) on the 10 couple-level variables representing husbands’ and wives’ scores on the 5 compensation subscales. We used a varimax rotation and an eigen-value criterion of 1, and the resulting 4-factor solution explained 72.10% of the variance. Factor loadings are presented in Table 3. Factor 1 included the husbands’ intra-individual strategies (Internal, Time, Effort) and Factor 2 included the wives’ intra-individual strategies (Internal, Time, Effort). Interestingly, husbands’ and wives’ External strategies loaded together on Factor 3, consistent with their correlation. Moreover, husbands’ and wives’ Reliance strategies loaded together on Factor 4. This further supports the view that, while partners’ intra-individual strategies were relatively independent of each other, their extra-individual strategies—particularly using external material resources but also relying on other people—were interdependent. This PCA suggests that, within couples, the use of both external and reliance strategies by each member tapped the same, couple-level construct.

**TABLE 3:** Factor loadings for wives’ (F) and husbands’ (M) compensation strategies, analysed at the couple level.

Subscale	Factor 1	Factor 2	Factor 3	Factor 4
F internal strategies	0.109	0.803	–0.096	0.126
F time strategies	–0.031	0.765	–0.035	0.029
F effort strategies	–0.007	0.798	0.330	–0.018
F external strategies	0.000	0.106	0.907	–0.053
F reliance strategies	–0.157	0.272	–0.048	0.809
M internal strategies	0.822	0.074	–0.080	–0.019
M time strategies	0.785	0.029	0.141	0.192
M effort strategies	0.852	–0.021	0.093	–0.057
M external strategies	0.130	–0.031	0.914	0.069
M reliance strategies	0.414	–0.158	0.078	0.663

*Shading illustrates how variables clustered into factors.*

To further understand the similarity or differences in external strategy use within couples, we examined relationships between scores for each of the different individual items on the External subscale. This subscale includes eight different items, asking about use of shopping lists, notebooks, bookmarks, books for recording phone numbers and birthdays, and organising the environment to support memory. Given these were single items and therefore scores were ordinal (i.e., restricted to a rating of 1–5), we obtained non-parametric Spearman’s correlations within couples on each item. This analysis indicated significant correlations between members of a couple for shopping lists, ρ = 0.33, *p* = 0.013, bookmarks when reading, ρ = 0.56, *p* < 0.001, posting notes on a board, ρ = 0.42, *p* = 0.001, putting things in particular places to remember them, ρ = 0.42, *p* = 0.002, writing appointments in a notebook or calendar, ρ = 0.74, *p* < 0.001, and writing phone numbers in a phone book, ρ = 0.44, *p* = 0.001. There were two External items that did not significantly correlate within couples; recording birthdays in a birthday book, ρ = 0.17, *p* = 0.223, and placing items by the door to be remembered when leaving the house, ρ = 0.25, *p* = 0.065. Interestingly, these latter two items were the only two that showed significant gender differences at the item level, such that non-parametric Wilcoxon Signed Rank tests indicated that women scored significantly higher than men, *p* = *p* < 0.001 and *p* = 0.002, respectively, all other tests *p* > 0.281. This suggests that some types of external memory compensation co-vary within couples, while for others, gender roles result in asymmetries within couples. We return to this point in the Discussion.

### “Memory Compensation Strategies in Daily Life” Interview

We conducted and coded 48 semi-structured interviews with the aim of understanding how couples individually and jointly used external memory tools in their day-to-day lives. We found that couples used a rich and complex array of material resources to support their remembering. Couples described a total of 113 regularly used memory resources across all interviews. Most commonly, couples reported having two material resources (19/48) to support their everyday remembering, but couples also variously reported using one (12/48), three (10/48), four (4/48), five (1/48), and even six (2/48) resources.

#### Type

Resources were most frequently physical rather than digital (100/113, 88.5%) and were more commonly fixed than portable (75/113, 66.4%) reflecting this older population’s relatively limited uptake of digital technology. Smartphones, for instance, were rarely reported, and the most common external memory resources were prominently displayed calendars and diaries.

#### Function

Overwhelmingly, external memory resources were used to support “prospective memory” [i.e., events in the future; see Browning et al. (2018)]: 86/113 resources (76.1%) were used to remember appointments and upcoming tasks. More rarely, 13/113 (11.5%) resources were used for “autobiographical memory” [i.e., personally experienced life events, see Harris et al. (2014)], including journals and photo albums. Some resources were used for both kinds of remembering: 14/113 resources (12.4%) were diaries or similar in which daily tasks were recorded but which were also stored (sometimes for decades) for reminiscing purposes:

*F: We try and put everything on the calendar…it’s a good way of remembering. If there’s anything important, we can just look back.*

Interestingly, the MCQ items regarding external resources all refer to prospective memory rather than to episodic or autobiographical remembering, consistent with the way the items were generated by individuals reflecting on their most common everyday memory tasks. Our interview data suggest that external material resources are also used, albeit less frequently, for autobiographical remembering, and that some resources are initially used for prospective remembering but later stored for autobiographical record-keeping.

#### Coordination

Couples reported complex and varied ways in which they coordinated their remembering practices, and particularly their external resource use. Some couples maintained separate diaries or calendars, but would explicitly and reciprocally update one another so that their resources reflected the same information:

*F: We need to have a diary session every couple of months so we’re not double-booking each other.*

Other couples used diaries and calendars as a proxy to check a partner’s availability, or remind them of an upcoming event, in the case that they were not home:

*M: If [wife’s] not here I can go to the calendar and see if she’s tied up on a particular day, and vice versa.*

In this way, external memory resources were an extension of the in-person communication couples employed in coordinating their shared day-to-day lives. Couples often had enduring systems in place of which both partners were aware:

*F: We share this diary, so we put both our regular events in it.*

*M: I put mine in red on one side, and [wife] puts hers in pencil on the other.*

Couples coordinated their memory compensation strategies to jointly assist them in accomplishing the memory tasks of daily life. Particularly evident was the extent of interaction and coordination between social and material memory supports, underscoring how couples’ cognitive processes involve complex entanglements with both and material aspects of their environment.

#### Responsibility

Shared resources were most commonly reported, but closer analysis of the interview data revealed that responsibility for maintaining and checking memory resources was unevenly distributed along gendered lines. For the 12/48 couples who reported a single resource, most (11/12) described it as a shared resource, but 3/12 were only checked by the women, 3/12 were only written in by the women, 6/12 reported that the husband at least sometimes used the resource “vicariously” *via* the wife, while only 1/12 reported that both partners engaged in vicarious use.

Averaged across the sample, it was most common for resources to be considered “shared” by both members of the couple (52.1%), or owned by the wife (27.4%), compared to owned by the husband (20.4%). Similar patterns were evident for checking of resources: 46.9% of resources were checked by both partners, 32.7% were checked only by the wife, and 20.4% were checked only by the husband. This gender disparity was more prominent when it came to entering details into resources: 45.1% of resources were maintained by both partners, while 35.5% were maintained solely by the wife, and 19.5% were maintained solely by the husband.

While women tended to take greater responsibility for the checking and maintenance of memory resources in general, analysis of the interviews revealed that couples had idiosyncratic ways of dividing responsibility when it came to remembering. For example, some couples used a shared resource where the wife might be responsible for entering social events and birthdays, while the husband would use the same resource to note the due date of an electricity bill:

*F: [Husband] does things in the diary. He does not do appointments and outings, but he writes things like when a bill comes in.*

*M: I diarise bills coming in.*

Responsibility could also be divided between the maintenance and the checking of memory resources. For example, it was quite common for wives to be largely responsible for entering appointments into a diary or a calendar that their husband had a daily ritual of checking.

*F: Appointments are written down in the diary. Everyone’s birthday, anniversaries, everything’s written down. And [husband] is my secretary, he looks at it a lot of times.*

Therefore, using shared systems and resources was often beneficial as it meant that information entered by one partner could be accessed by both.

#### Vicarious Benefit

Such benefits were not always evenly distributed, as it was frequently the case that one partner held greater responsibility for the maintenance of resources (usually the wife), from which their partner vicariously benefited either by checking or through explicit reminders from their partner. Of the 113 resources described, 43 (38%) had some evidence of “vicarious” use, in which one person accessed the resource *via* their spouse, as exemplified in the following three quotes:

*M: I do not use the calendar, but [wife] refers to it constantly, and she’ll remind me.*

*M: No, I do not use memory aids … Oh hang on, [wife] carries a diary with her all the time. … Oh, well, if she’s got the diary, we’re always together and that’s it.*

*M: I rely on [wife’s] diary. I do not have a diary myself, and everyone says you should have a diary, but [wife] has a diary, and she’s got all the things down.*

Men were far more likely to benefit from vicarious resource use in this way: we scored 30 resources from which the husband vicariously gained, compared to only seven for wives. In these instances, each of the men scored low on questionnaire measures of external memory compensation strategies, but these comments indicated that he was in fact using and benefitting from external tools and resources, in communication and consultation with his wife.

#### Reliance

A common theme among our interviews was the importance that couples placed on their external memory resources, to reduce the cognitive burden of remembering in day-to-day life:

*F: We write down anything vital so we do not have to [remember it]. We do not rely on our brains, do not need to be fogged up with all that detail.*

Couples also recognised the ways that they relied on one another to support memory, in tandem with other resources. Evident in these interviews was the implicit and automatic nature of the social memory support that members of these couples provide for one another. One man commented while completing the MCQ questionnaire, to explain his lack of strategy use:

*M: I do not have to remember. My wife’s good—oh, god, she’ll tell you all the telephone numbers of all the kids and all the rest of it, but I … do not have to.*

While Reliance strategies are consistently rated the lowest on the MCQ, this may be because they ask for a very deliberate and explicit seeking of social support “do you ask someone to help you,” while the memory support that comes from a longstanding transactive memory system is likely to be ever-present and implicit, rather than sought out. As one couple explained, when asked how they remembered together:

*F: It’s sort of grown over the decades and we’re not aware of what we do.*

*M: Well, we’ve spent a lot of time together.*

#### Age-Related Memory Decline

Another common theme among our interviews was the increasing importance of memory supports (both social and material) in the face of perceived age-related cognitive decline. Several couples spoke about an increased need for discipline in using external memory resources:

*F: We use the calendar a lot more frequently. Before we did not, we remembered it, but now we make sure it’s on the calendar—everything.*

*M: It’s accommodation to the fact that your memory is deteriorating. You’ve got to develop a technique to get round the problems that that causes.*

It was evident from these interviews that external memory resources, and the established routines and systems around their shared use, are particularly important for supporting couples’ remembering as they age.

Our rich interview data complemented the questionnaire-based data. Couples’ cognitive interdependence was evident not only in their similar reporting of memory compensation strategies, but also in the ways they coordinated these strategies with each other, and the way that they established their environments, their lives and routines, and their broader systems for remembering. These complex and idiosyncratic systems involved integrated material and social memory supports, developed over decades of shared experience and highly interconnected lives.

## Discussion

We examined interdependence in the reported memory compensation strategies of 116 individuals who made up 58 older, long-married couples, using both an established questionnaire measure of memory compensation as well as an open-ended interview. Consistent with prior research (Dixon et al., 2001), we found that external strategies—use of diaries, calendars, and other physical tools—were the most frequently reported, and that reliance on one’s spouse was least frequently reported, even in this population who were all in intimate, longstanding marital relationships. However, women scored higher than men on reported external strategies, and men higher than women on reliance strategies. While the intra-individual strategies of husbands and wives loaded on distinct factors, their use of external strategies and reliance strategies loaded together indicating that they accounted for shared variance. These results imply that there are interrelationships within the memory compensation strategies used by individuals within longstanding couples, and particularly there are relationships in their use of extra-individual strategies, whether material or social.

Our findings provide support for a conceptualisation of longstanding intimate couples as interdependent cognitive systems (see also Dixon, 1999; Harris et al., 2014), consistent with epidemiological research on shared cognitive and health trajectories within intimate dyads (Gerstorf et al., 2009; Hoppmann and Gerstorf, 2009, 2015; Hoppmann et al., 2011; Bourassa et al., 2015). Prior research has suggested that couples can remember more, and differently, when they collaborate than when they recall separately, such that they show “collaborative facilitation” (Gould and Dixon, 1993; Dixon and Gould, 1998; Harris et al., 2017; Barnier et al., 2018b). The performance benefits of remembering together depend critically on effective communication between partners (Gould et al., 1994; Harris et al., 2019), including on prospective memory tasks (Browning et al., 2018, 2019). Prior research has therefore examined couples’ cognitive interdependence *via* their joint memory task performance. The current findings add to this previous research by suggesting that couples coordinate their extra-individual memory compensation strategies, as they jointly engage in the memory tasks of daily life.

Specifically, we found a positive relationship within couples for use of external tools and resources to support memory, although this depended on the specific tool and memory task. Although we were interested in a range of possible interrelationships between couples’ responses, we found evidence of similarity in the extent of external strategy use. To use the language of transactive memory theory (Wegner et al., 1985), such similarity suggests “integration” in the system, although we note that a joint strategy of relying on external resources might be applied to quite differentiated domains of responsibility. We did also identify differentiation, particularly regarding division of responsibility according to gender roles. Overall, our results were consistent with a balance of integration and differentiation in effective transactive memory systems [see also Wegner et al. (1985); Wegner (1987), and Barnier et al. (2018a)]. They also suggest a potential distinction between the processes and contents of joint remembering, such that people within transactive memory systems may use similar processes to undertake differentiated tasks. Future research could address this distinction.

Our interviews yielded rich insights into the ways in which couples incorporate external memory compensation strategies into their homes and joint lives, emphasising potential interrelationships between external and reliance strategies in these kinds of intimate groups. Couples’ systems for memory compensation were complex and diverse, with a range of different ways of sharing and coordinating cognitive labour. These complexities are often not captured by theories and measures that index different kinds of compensation strategies in isolation from each other. Future research could extend measures of memory compensation strategies to ask about interaction or blending between social and material strategies, as well as vicarious use of memory compensation strategies. The present study paves the way for research on the interaction between different kinds of external memory supports—particularly how the social and material interact—perhaps especially in the context of older long-term couples who may be (individually) experiencing memory decline and increasingly benefit from external strategies (Dixon, 2011). Overall, our findings support conceptualizing intimate couples as interdependent when assessing their memory performance, their practices, and their everyday memory functioning, such that performance and function are better understood when studying individuals in the context of their memory supports, including their partner (c.f. Harris et al., 2014).

We also found pervasive gender differences in both the questionnaire and interview data. Women used more External strategies and men used more Reliance strategies, consistent with previous research (Dixon et al., 2001). We found that concern about memory success increased the use of internal strategies for both men and women, and increased the use of Reliance strategies for men only. Moreover, we found evidence that men frequently benefitted from vicarious use of external resources maintained by their wives. The results are consistent with broader research on gender and cognitive labour, with social research finding that women take disproportionate responsibility for day-to-day cognitive labour within heterosexual relationships (Ahn et al., 2017). Moreover, recent research found that men underperformed on a prospective memory task compared to women (post a letter to the researchers each day for 7 days), but only for those in relationships (Niedźwieńska and Zielińska, 2020), such that being in a heterosexual partnership was associated with increased prospective memory performance for women and decreased prospective memory performance for men. Understanding these gender differences in memory compensation are important, as they suggest that gender may impact on cognitive trajectory over the lifespan and adaptation to risk factors and adverse life events, both normative and non-normative.

We did note several limitations with the memory compensation questionnaire that present avenues for further conceptual development and empirical research. The MCQ was developed by surveying large numbers of older adults about the memory tasks they complete in day-to-day life, and the compensation strategies they use. These items were refined to load on to distinct factors, capturing distinct types of compensation (de Frias and Dixon, 2005). In the current research, when combining questionnaire and interview data, we noted that there were interactions between different strategies. This was particularly evident in the ways that reliance on social supports could involve the vicarious use of other strategies especially external strategies, such that a focus only on the individual could underestimate the ways in which they were benefitting from memory compensation strategies in their broader transactive memory system. We also noted that exemplar memory tasks used in the MCQ items refer to both prospective and episodic memory, consistent with the tasks generated by older adults when the questionnaire was first established. However, these memory tasks types are unevenly split across strategies, with internal strategies most often rated in terms of remembering the past and both external and reliance strategies most often rated in terms of remembering a future task or appointment. This asymmetry may match actual use in everyday life, and future research could examine this by counterbalancing the task type to determine whether different strategies are more often invoked for different memory tasks. We note that the MCQ strategy subscales varied in whether they referred to strategies that operated to enhance encoding vs. retrieval of information. Again, future research is needed to examine whether internal and external strategies can be effectively employed at both encoding and retrieval.

Finally, we note that both the MCQ and our interview were limited to asking people about the memory practices that they were aware of and could consciously reflect upon and describe. We asked people to describe their remembering practices, rather than to remember, as such. This may have biased people to consider and report on particular kinds of tasks that they most immediately thought of as memory tasks. More implicit and automatic interactions with ever-present and familiar aspects of the environment, such as photos and memorabilia, may have been neglected because of this approach (Habermas and Paha, 2002; Chapman, 2006). Future research could examine the way that couples independently and jointly recall in response to these kinds of object-based cues. Indeed such object-based memory cues may be particularly important for supporting autobiographical memory in the face of cognitive decline (Kirk et al., 2019).

Overall, we found evidence of interdependence in the memory compensation strategies adopted by older adults within long-married couples, as well as interaction between social and material resources that couples use to support memory in day-to-day life. Conceptualising couples as dynamic and interactive systems and investigating the shared and coordinated nature of memory compensation emphasises the role of the broader social context in determining how people support their memory performance as they age together with their spouse. Future research is needed to directly test the impact of memory compensation on memory performance. Alongside the theoretical implications, our findings have practical implications for memory and ageing. We argue that recommendations for utilising memory compensation strategies as a form of functional rehabilitation for cognitive decline could focus more directly on dyads, and on encouraging people within couples to jointly adopt and coordinate memory compensation strategies, recognising that cognitive changes in one individual impact on the couple as a whole [see also Dixon (2011, 2013)]. Conceptualising people’s cognition as existing within an ecological framework of social and material supports is likely to have implications for assessing individuals’ everyday competence, by which individuals may function very effectively when surrounded by a supportive environment of familiar tools and strategies, despite showing impairments on more clinical measures (c.f. Dixon, 2011, 2013).

## Data Availability Statement

The raw data supporting the conclusions of this article will be made available by the authors, without undue reservation.

## Ethics Statement

The studies involving human participants were reviewed and approved by Macquarie University Human Research Ethics Committee. The patients/participants provided their written informed consent to participate in this study.

## Author Contributions

CH, JS, and RD conceived the study. CH, PK, AB, and GS were involved in data collection. CH analysed the quantitative data and drafted the manuscript. CH and JS developed the coding system for the interview data. NM and SH analysed the interview data. All authors discussed the findings and interpretation and reviewed and finalised the manuscript.

## Conflict of Interest

The authors declare that the research was conducted in the absence of any commercial or financial relationships that could be construed as a potential conflict of interest.

## Publisher’s Note

All claims expressed in this article are solely those of the authors and do not necessarily represent those of their affiliated organizations, or those of the publisher, the editors and the reviewers. Any product that may be evaluated in this article, or claim that may be made by its manufacturer, is not guaranteed or endorsed by the publisher.
